# Comparative Transcriptome Analysis of Different *Dendrobium* Species Reveals Active Ingredients-Related Genes and Pathways

**DOI:** 10.3390/ijms21030861

**Published:** 2020-01-29

**Authors:** Yingdan Yuan, Bo Zhang, Xinggang Tang, Jinchi Zhang, Jie Lin

**Affiliations:** 1 Co-Innovation Center for Sustainable Forestry in Southern China, Nanjing Forestry University, Nanjing 210037, China; yuanyingdan@126.com (Y.Y.); bozhangophelia@gmail.com (B.Z.); xinggangtang@126.com (X.T.); 2 Jiangsu Province Key Laboratory of Soil and Water Conservation and Ecological Restoration, Nanjing Forestry University, Nanjing 210037, China; 3 Department of Environmental Science and Policy, University of California, Davis, CA 95616, USA

**Keywords:** *Dendrobium*, comparative transcriptome, active ingredients, different tissues

## Abstract

*Dendrobium* is widely used in traditional Chinese medicine, which contains many kinds of active ingredients. In recent years, many *Dendrobium* transcriptomes have been sequenced. Hence, weighted gene co-expression network analysis (WGCNA) was used with the gene expression profiles of active ingredients to identify the modules and genes that may associate with particular species and tissues. Three kinds of Dendrobium species and three tissues were sampled for RNA-seq to generate a high-quality, full-length transcriptome database. Based on significant changes in gene expression, we constructed co-expression networks and revealed 19 gene modules. Among them, four modules with properties correlating to active ingredients regulation and biosynthesis, and several hub genes were selected for further functional investigation. This is the first time the WGCNA method has been used to analyze *Dendrobium* transcriptome data. Further excavation of the gene module information will help us to further study the role and significance of key genes, key signaling pathways, and regulatory mechanisms between genes on the occurrence and development of medicinal components of *Dendrobium*.

## 1. Introduction

*Dendrobium* is a perennial epiphytic herb of the genus *Orchidaceae*, with more than 1500 species in the world which are mostly growing in tropical and subtropical Asia and eastern Australia [1,2]. In China, there are more than 80 species of *Dendrobium* that had been reported in studies, mainly distributed in south of the Qinling Mountains [3,4]. As a kind of ornamental and medicinal plants, more and more researchers have paid attention to it in recent years [5]. Also, *Dendrobium* is a traditional Chinese herbal medicine, that has been used for benefiting stomach and clearing heat, nourishing yin, and promoting fluid [6]. The medicinal ingredients of *Dendrobium* are very complex, not only including polysaccharides and alkaloids, but also flavonoids, various amino acids, and trace mineral elements. However, the most two important components are polysaccharides and alkaloids [7,8,9,10].

With the development of high-throughput technology, a variety of data sources have been derived, including gene expression microarray, RNA-seq, metabolomic and CHIP-seq, these have become powerful tools for studying plant growth, development, and physiology at the transcriptional and metabolic levels [11,12,13]. In order to explore the key genes and regulatory pathways for the synthesis of its medicinal ingredients, more researchers have done different kinds of *Dendrobium* transcriptome research [14,15,16]. However, it has been difficult to systematically explain the relationship between gene expression or metabolite changes and trait differences [17]. The analysis of correlation networks can bridge the gap between single gene interpretation and systematic biology research by mining the link between genes and gene products [18,19,20], such as integrating a single gene into a co-expression network based on pairwise gene expression correlation [21]. Gene co-expression analysis has been used to discover new candidate genes [22,23], identify key modulators of immune responses [24], and reconstruct regulatory pathways [25]. Genes belonging to the same co-expression sub-network (or module) are likely to be functionally related [26,27,28], participate in similar biological processes, or be part of the same pathway [25].

Weighted gene co-expression network analysis (WGCNA) is one of the most useful methods based on gene co-expression networks. It focuses on the set of genes other than on a single gene in the observed gene expression data, and it alleviates the multiple detection problems inherent in chip data analysis and can be used in unweighted correlation networks [29]. Compared with many other analysis methods, WGCNA has the advantages of summarizing and standardizing the methods and functions of integrated R packages, including methods of weighted and unweighted correlation networks [30]. WGCNA is used in combination with gene chip data, transcriptome data, and metabolome data for metabolic regulation network simulation, mining inter-genetic interactions, screening functional genes, etc. It has been extensively studied in plant growth, tissue and organ development, pigmentation and fragrance synthesis [31,32,33,34].

To reveal the underlying molecular mechanism of the active ingredients of *Dendrobium*, we downloaded three datasets from the NCBI Sequence Reading Archive (SRA) to identify highly connected hub genes and important modules. This study used three different *Dendrobium* species and different tissues as materials to perform transcriptome sequencing data, combined with polysaccharide and alkaloid content data, and used WGCNA analysis to construct a co-expression gene network. Correlation analysis was performed between the gene module and the polysaccharide and alkaloid data, and hub genes related to the main medicinal ingredients were discovered, in order to provide new clues for further research on the molecular mechanism of medicinal ingredients of *Dendrobium*. For the first time, a co-expression network analysis of transcriptome genes in *Dendrobium* was constructed, and modules with high correlation in secondary metabolism were analyzed, laying a foundation for the discovery of functional genes of medicinal ingredients.

## 2. Results

### 2.1. Determination of Total Alkaloid and Polysaccharide Contents in Different Species and Different Tissues

**Figure 1:**
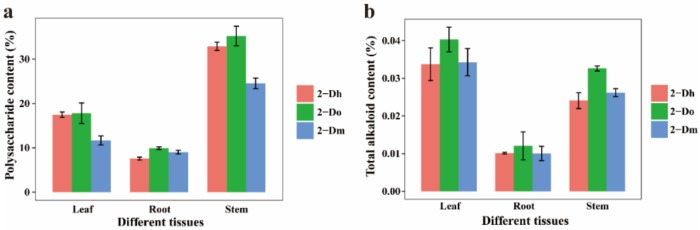
Polysaccharide and total alkaloid contents variation in different tissues of different species. (**a**) Polysaccharide content. (**b**) Total alkaloid content. There are three replicates of each sample. 2-Dh: two-year-old *Dendrobium huoshanese*, 2-Do: two-year-old *Dendrobium officinale*, 2-Dm: two-year-old *Dendrobium moniliforme*.

Polysaccharides and alkaloids are the main medicinal components of *Dendrobium*. Therefore, we determined the polysaccharide and total alkaloid in three different tissues of three *Dendrobium* species (Figure 1). The biennial *Dendrobium officinale* has the highest content in the comparison of different tissues and species. For polysaccharide content, the stem is the main enrichment tissue, while for total alkaloid, the leaf is the main enrichment tissue. In the stem, the polysaccharide content of *Dendrobium* varied from 23.34 to 37.41%. In the leaf, the total alkaloid content of *Dendrobium* varied from 0.0291 to 0.0421%.

### 2.2. Differential Expression Analysis

**Figure 2:**
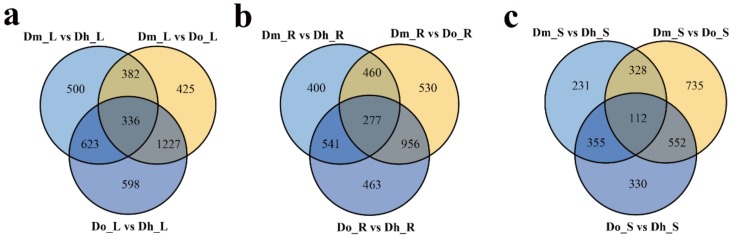
Venn diagram of differentially expressed genes (DEGs) in different comparisons. All DEGs are grouped into three comparison groups represented by three circles. The overlapping portions of the different circles represent the number of DEGs common to these comparison groups. (**a**) Venn diagram of three kinds of *Dendrobium* leaves. (**b**) Venn diagram of three kinds of *Dendrobium* roots. (**c**) Venn diagram of three kinds of *Dendrobium* stems.

**Figure 3:**
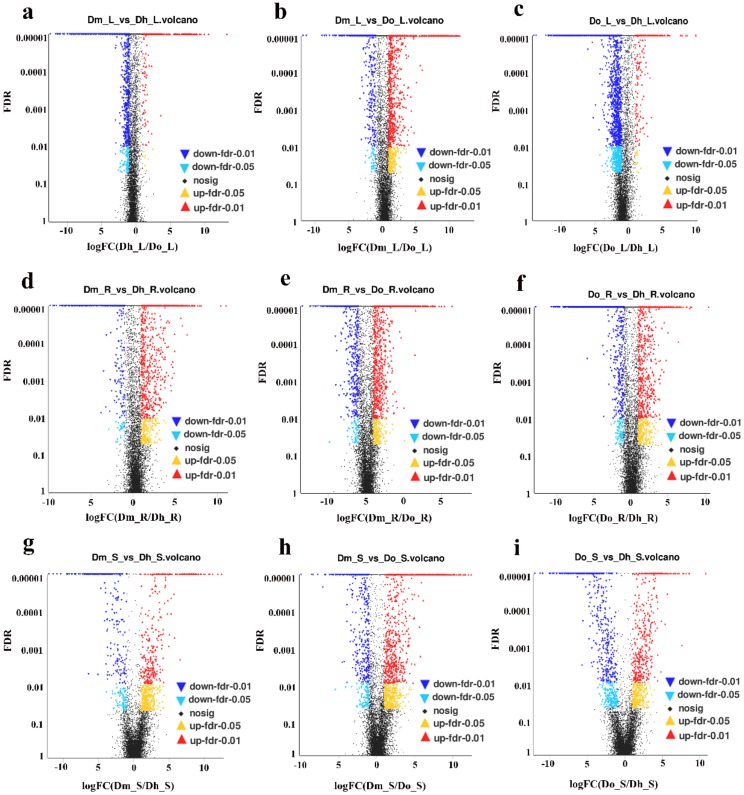
Volcano plots of the DEGs in different comparisons. Red dots indicate significant up-regulation of genes, and blue dots indicate significant down-regulation of genes. Black dots represent non-DEGs. (**a**) Dm_L vs. Dh_L volcano; (**b**) Dm_L vs. Do_L volcano; (**c**) Do_L vs. Dh_L volcano; (**d**) Dm_R vs. Dh_R volcano; (**e**) Dm_R vs. Do_R volcano; (**f**) Do_R vs. Dh_R volcano; (**g**) Dm_S vs. Dh_S volcano; (**h**) Dm_S vs. Do_S volcano; (**i**) Do_S vs. Dh_S volcano.

In this study, we calculated the FPKM of each sample to regulate the expression and investigate the gene expression differences among different tissues of different *Dendrobium* species. We compared the three data sets from different comparison groups using a Venn diagram (Figure 2a–c). In addition, we compared the number of DEGs between different species in the same tissue, and many genes were differentially expressed only in one or two comparisons. Then the edgeR was used to test the differential expression of the repeated count data. We used “FDR < 0.05 & | log_2_FC | ≥1” as the criterion for significant differences in gene expression. When log_2_FC > 1, DEG is considered to be up-regulated. In contrast, for log_2_FC < −1, it is considered a downward adjustment. We did up and down-regulated analysis of DEGs for three different tissues and three different *Dendrobium* species, and the results are shown in Figure 3a–i.

**Figure 4:**
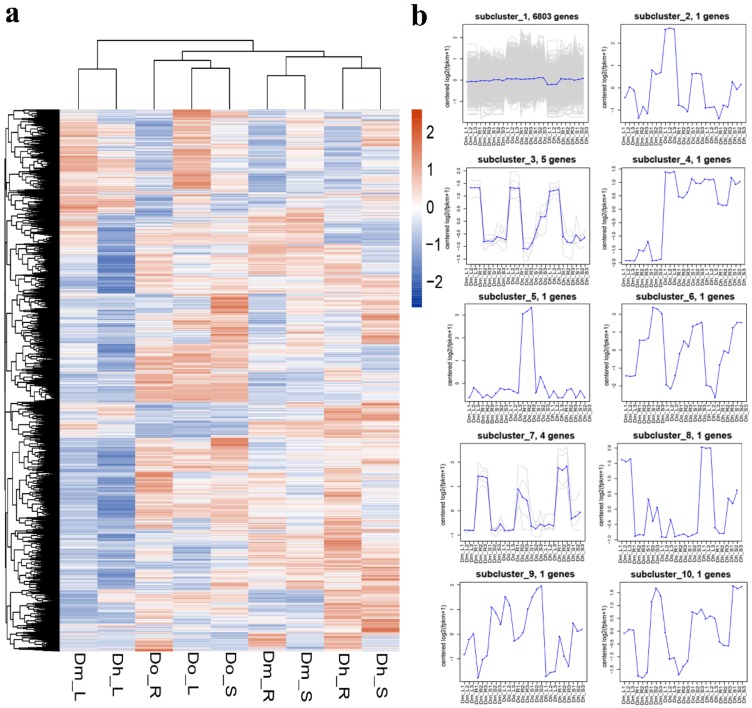
Expression profiles of all DEGs. (**a**) The heatmap of DEGs and the FPKM distribution of all unigenes obtained by hierarchical cluster analysis. Each column in the figure represents a sample, and each row represents a gene. The colors in the graph indicate the magnitude of gene expression (log_10_ (FPKM + 1)) in the sample. Red indicates that the gene is highly expressed in the sample, and the blue indicates that the gene expression is low. (**b**) K-means clustering analysis of gene expression profiles. The blue line represents the expression model. The gray lines are the expression profiles of each DEGs. The x-axis represents different tissues of different Dendrobium plants. The y-axis represents log_2_ (ratio).

According to the transcriptome data, all DEGs were used for hierarchical cluster analysis of transcription abundance in three different tissues. The heatmap of DEGs between different tissues and different *Dendrobium* species show similar transcriptome profiles for Dh_R, Dh_L, Dh_S, Do_R, Do_L, Do_S, Dm_R, Dm_L, and Dm_S (Figure 4a). A total of 35,159 DEGs were identified and analyzed using criteria of log_10_ (FPKM+1) and *p* < 0.05. The trend of the specific expression level is shown in the number under the color bar at the top left. On the left is the gene cluster tree. The closer the degree of separation between the two genes, the closer their expression is. In order to reflect the main trends and tissue-specific expression of different *Dendrobium* species, all DEGs were clustered into ten expression profiles (Figure 4b) using the K-means method and hierarchical clustering with similar regulation model and log_2_ (foldchange). DEGs belonging to cluster 3 were more highly expressed in leaves than in other tissues. Except for subcluster 1 and subcluster 3, all the other subclusters have just one gene trend.

### 2.3. GO and KEGG of DEGs Annotation and Enrichment Analysis

**Figure 5:**
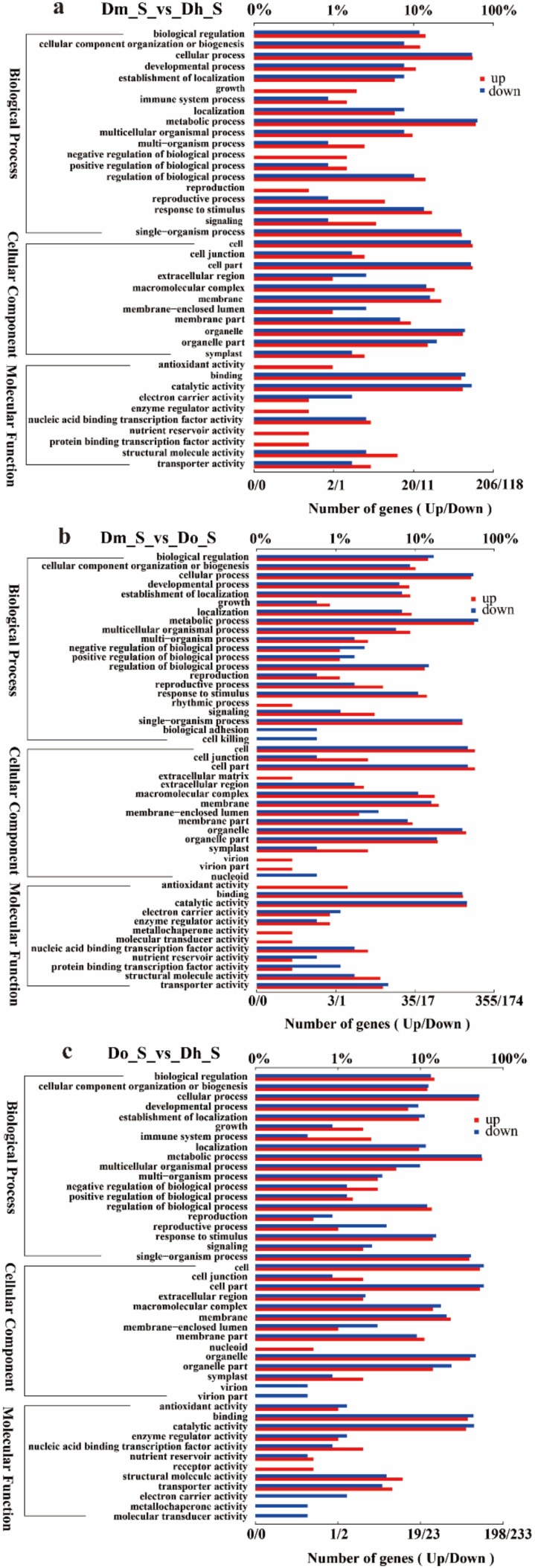
Gene Ontology (GO) annotations of up and down regulated DEGs. The bottom x-axis indicates the number of DEGs annotated to a GO term, the upper x-axis indicates the proportion of DEGs annotated to a GO terms to the total number of all GO annotated DEGs; and the y-axis represents each detailed classification of GO. (**a**) Dm_S vs Dh_S GO annotation; (**b**) Dm_S vs. Do_S GO annotation; (**c**) Do_S vs. Dh_S GO annotation.

GO is a database established by the Gene Ontology Consortium. Its purpose is to standardize biological terms about genes and gene products in different databases, to define and describe gene and protein functions. Using the GO database, genes can be classified according to biological process, cellular component, and molecular function. The GO annotation statistics were performed on the differentially expressed genes in pairs, and one of the samples was used as a control. The obtained results can be used to plot the GO annotation bar graph of up and down DEGs (Figure 5). Figure 5 only shows the comparison of the stems of different *Dendrobium* species, and the other two tissues are shown in the Supplementary Figure S1.

**Figure 6:**
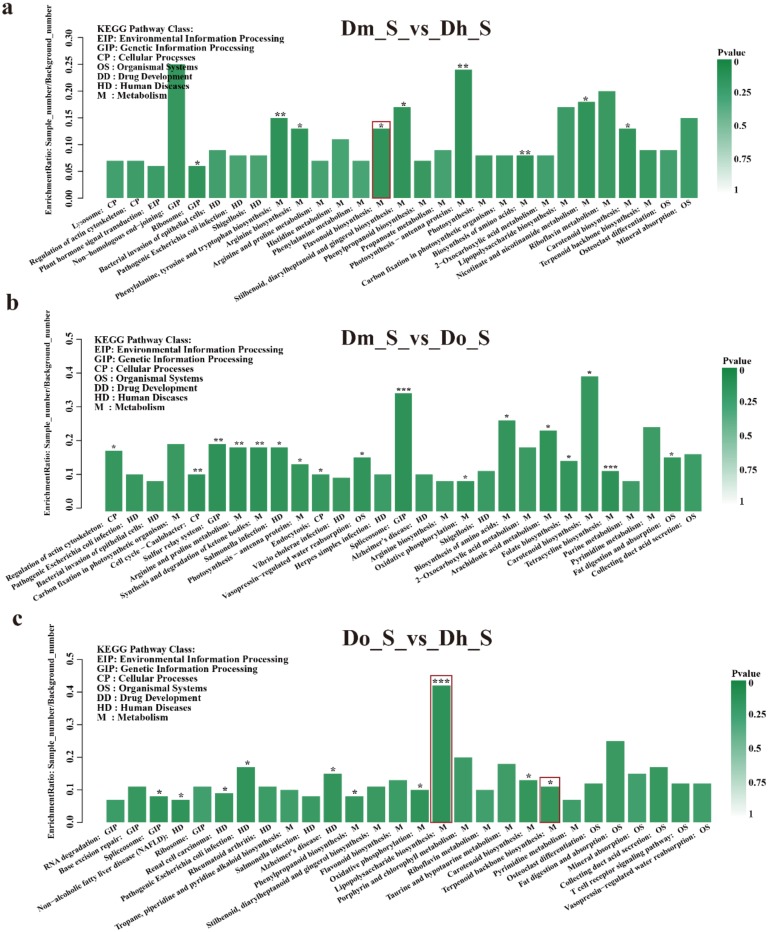
Kyoto Encyclopedia of Genes and Genomes (KEGG) pathway enrichment of DEGs. The x-axis represents the pathway name, and the y-axis represents the enrichment ratio (sample number/background number). (**a**) Dm_S vs. Dh_S; (**b**) Dm_S vs. Do_S; (**c**) Do_S vs. Dh_S. All pathways in the figure with asterisks indicate significant KEGG enrichment, with three asterisks indicating *p*-value < 0.001, two asterisks indicating *p*-value < 0.01, one asterisks indicating *p*-value < 0.05.

KEGG focuses on biochemical pathways, especially genes involved in protein, carbohydrate, and energy metabolism. In the KEGG enrichment analysis, we mapped all these genes to a reference pathway in the KEGG database to determine the biological pathways in which these genes may be involved (Figure 6). In terms of the KEGG pathways, “Dm_S vs. Dh_S” comparisons are involved in 235 pathways (Figure 6a). In “Dm_S vs. Do_S” comparisons, 223 pathways were involved (Figure 6b). Finally, in “Do_S vs. Dh_S” comparisons, 237 pathways were involved (Figure 6c). Among these pathways, “flavonoid biosynthesis” was enriched in the Dm_S vs. Dh_S comparison. “Lipopolysaccharide biosynthesis” related to polysaccharide was the most enriched pathways in the Do_S vs. Dh_S comparison. Notably, we observed terpenoid biosynthesis in the secondary metabolites also enriched in the Do_S vs. Dh_S comparison. The KEGG pathways of different *Dendrobium* species of the other two tissues are shown in the Supplementary Figure.

### 2.4. WGCNA Analysis

#### 2.4.1. Construction of Gene Co-Expression Network of Dendrobium

**Figure 7:**
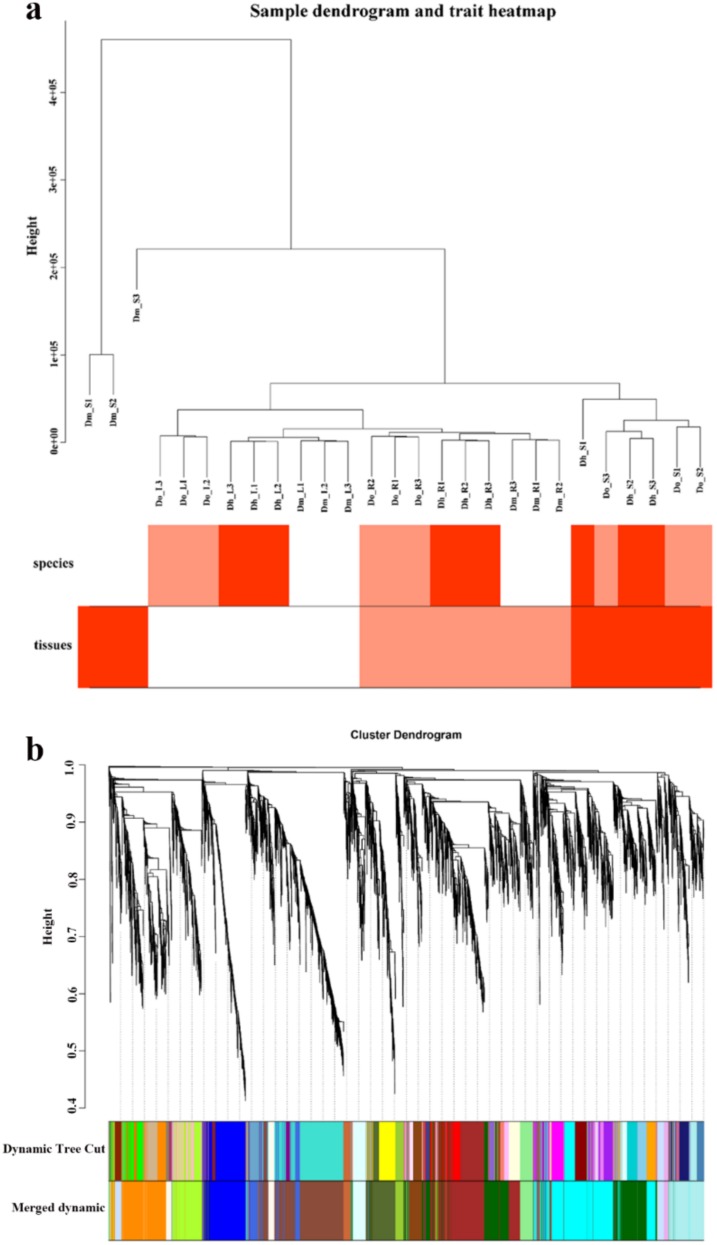
Clustering dendrogram. (**a**) Clustering dendrogram of 27 samples and heatmaps of species and tissues traits. The expression is from low to high, and the color transitions from white to red. (**b**) Clustering dendrogram of DEGs, with dissimilarity based on the topological overlap, together with assigned module colors. The clustered branches represent different modules, and each line represents one gene.

Consequently, we used the WGCNA package tool to construct a co-expression module that expressed the expression of 8056 genes in 27 *Dendrobium* samples. The heat map of the cluster dendrogram and tissues and species traits are shown in Figure 7a. One of the most critical parameters is the power value, which mainly affects the independence and average connectivity of the co-expression modules. First, we selected the appropriate power value. When the power value was 18, the independence reached 0.6, and the average connectivity was higher. Therefore, the power values and results of constructing co-expression modules indicate that 19 different co-expression modules were identified in *Dendrobium*. These co-expression modules were constructed and are shown in different colors (Figure 7). The size of these modules depends on the number of genes they contain. The number of genes and module names are shown in Table 1.

#### 2.4.2. Interaction Analysis of Co-Expression Module

**Figure 8:**
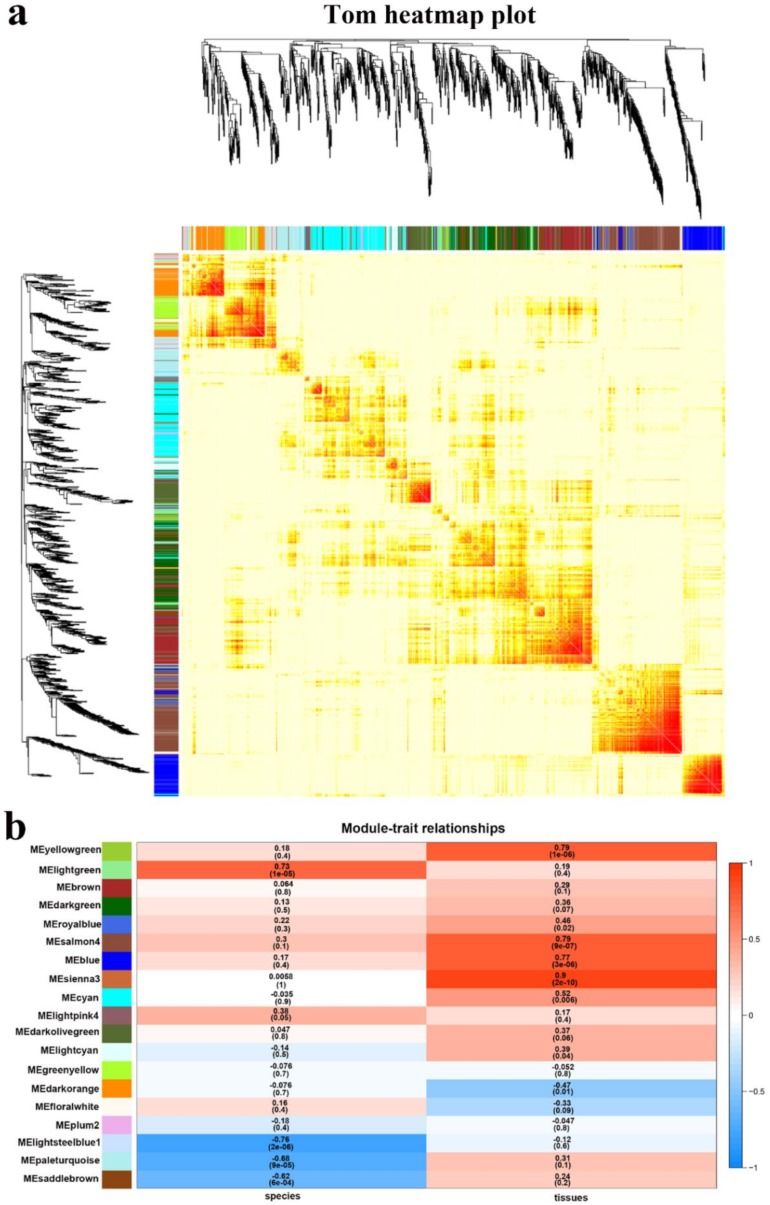
Co-expression network analysis across different tissues and different species. (**a**) Visualizing the gene network using a heatmap plot. The heatmap depicts the topological overlap matrix (TOM) among all genes in the analysis. (**b**) Module-trait associations. Each row corresponds to a module characteristic gene (eigengene), and each column corresponds to a trait. Each cell contains a corresponding correlation and *p*-value. According to the color legend, the table is color-coded by correlation.

Subsequently, we analyzed the interactions between the 19 co-expression modules (Figure 8). The heatmap shows the topological overlap matrix (TOM) of all genes in the analysis. Light color indicates low overlap, and dark red indicates high overlap. Except for some high-brightness areas, the overall difference between the modules is not significant, which indicates that the gene expression between the modules is relatively independent and has a high scale independence. The correlations between module eigengene and traits: tissues and species, were analyzed and data are shown in Figure 8b. Two modules are significantly associated with species: Lightgreen (*p*-value = 1 × 10^−5^, cor = 0.73) and Lightsteelblue1 (*p*-value = 2 × 10^−6^, cor = −0.76). The modules that highly correlated to tissues were Yellowgreen (*p*-value = 1 × 10^−6^, cor = 0.79), Salmon4 (*p*-value = 9 × 10^−7^, cor = 0.79), Blue (*p*-value = 3 × 10^−6^, cor = 0.77), and Sienna3 (*p*-value =2 × 10^−10^, cor = 0.9).

**Figure 9:**
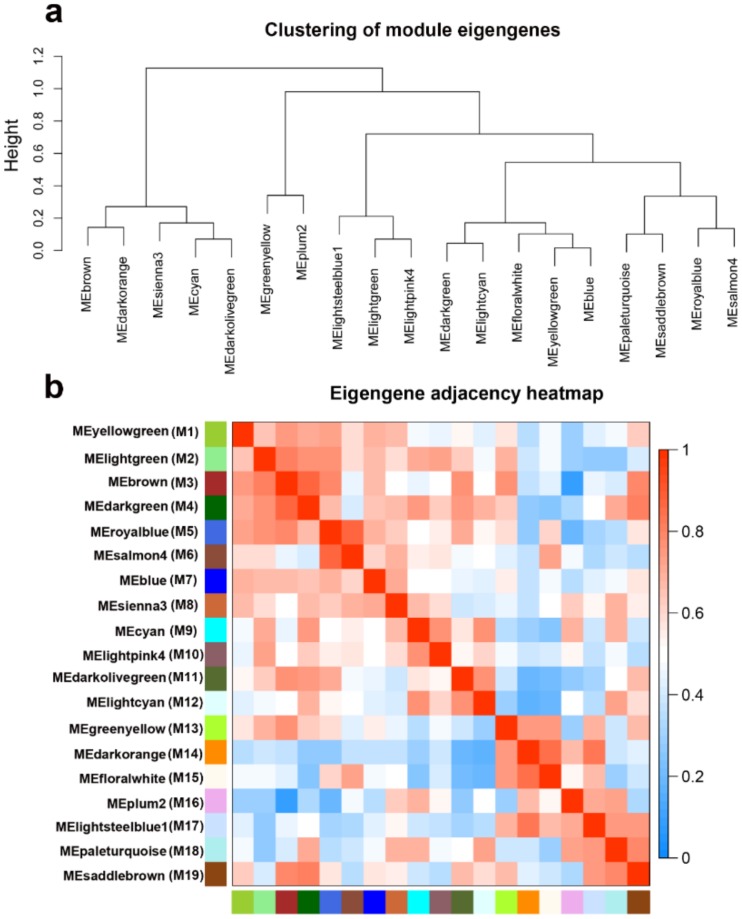
Analysis of connectivity of eigengenes in different module. (**a**) Cluster analysis of eigengenes. (**b**) The heatmap of connectivity of eigengenes.

We analyzed the connectivity of eigengenes to find the interactions between these constructed co-expression modules. First of all, cluster analysis of these eigengenes was performed (Figure 9a). These 19 clusters were divided into two clusters, including 5 modules (modules 3, 8, 9, 11, and 14) and the remaining 14 modules. According to that, the connectivity effect between different modules is obviously different. (Figure 9b).

#### 2.4.3. Functional Enrichment Analysis of Genes in Interested Modules

**Figure 10:**
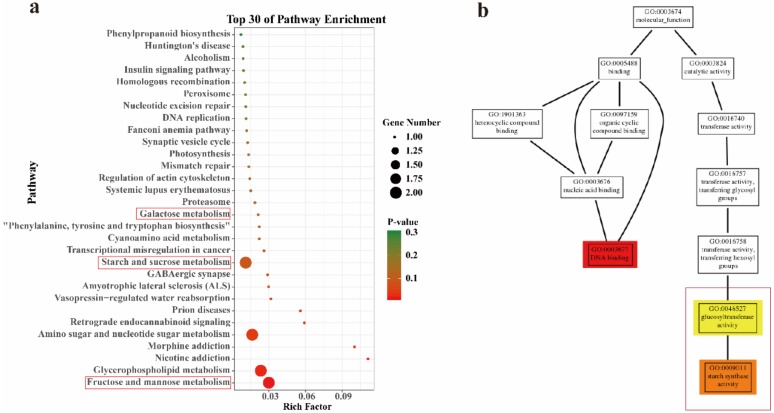
GO and Kyoto Encyclopedia of Genes and Genomes (KEGG) pathway enrichment of module sienna3. (**a**) Top 30 of KEGG pathway enrichment. (**b**) Thumbnails view of directed acyclic graph (DAG) on molecular function of module sienna3.

We performed pathway enrichment analysis on the genes of each module separately, explored their related functions, and obtained many meaningful KEGG pathway and GO terms (Figure 10). It is important to identify the most significant modules related to medicinal components. Sienna3 module showed a significantly high correlation with polysaccharide. In the KEGG pathway, it is significantly enriched to fructose and mannose metabolism (ko00051), starch and sucrose metabolism (ko00500), and galactose metabolism (ko00052). The top three GO terms, shown in expanded size, are glucosyltransferase activity (GO:0046527) and starch synthase activity (GO:0009011). These KEGG pathways and GO terms are all related to polysaccharide biosynthesis, and polysaccharides are the main medicinal components of *Dendrobium*.

**Figure 11:**
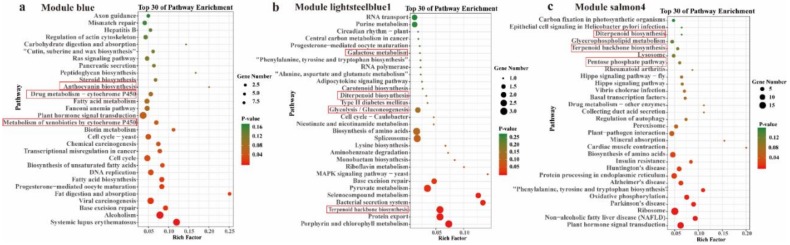
Kyoto Encyclopedia of Genes and Genomes (KEGG) pathway enrichment of interested module. (**a**) Module blue; (**b**) module lightsteelblue1; (**c**) module salmon4.

In addition, the KEGG pathways regulating *Dendrobium* polysaccharides and secondary metabolism biosynthesis were also identified in three other modules, which are the blue module, the lightsteelblue1 module, and the salmon4 module (Figure 11). In blue module, genes were significantly enriched in these pathways: anthocyanin biosynthesis (ko00942), drug metabolism—cytochrome P450 (ko00982) and steroid biosynthesis (ko00100). In lightsteelblue1, the significantly enriched pathways are as follows: terpenoid backbone biosynthesis (ko00900), diterpenoid biosynthesis (ko00904), galactose metabolism (ko00052), and glycolysis/gluconeogenesis (ko00010). In salmon4, genes were significantly enriched in these pathways: the pentose phosphate pathway (ko00030), terpenoid backbone biosynthesis (ko00900), and diterpenoid biosynthesis (ko00904). The enriched pathways in these three modules are significantly related to the synthesis of polysaccharides and secondary metabolites of *Dendrobium*. Therefore, sienna3, blue, lightsteelblue1 and salmon4 modules were defined as important module and extracted for further analysis.

#### 2.4.4. Module Visualize and Hub Genes

**Figure 12:**
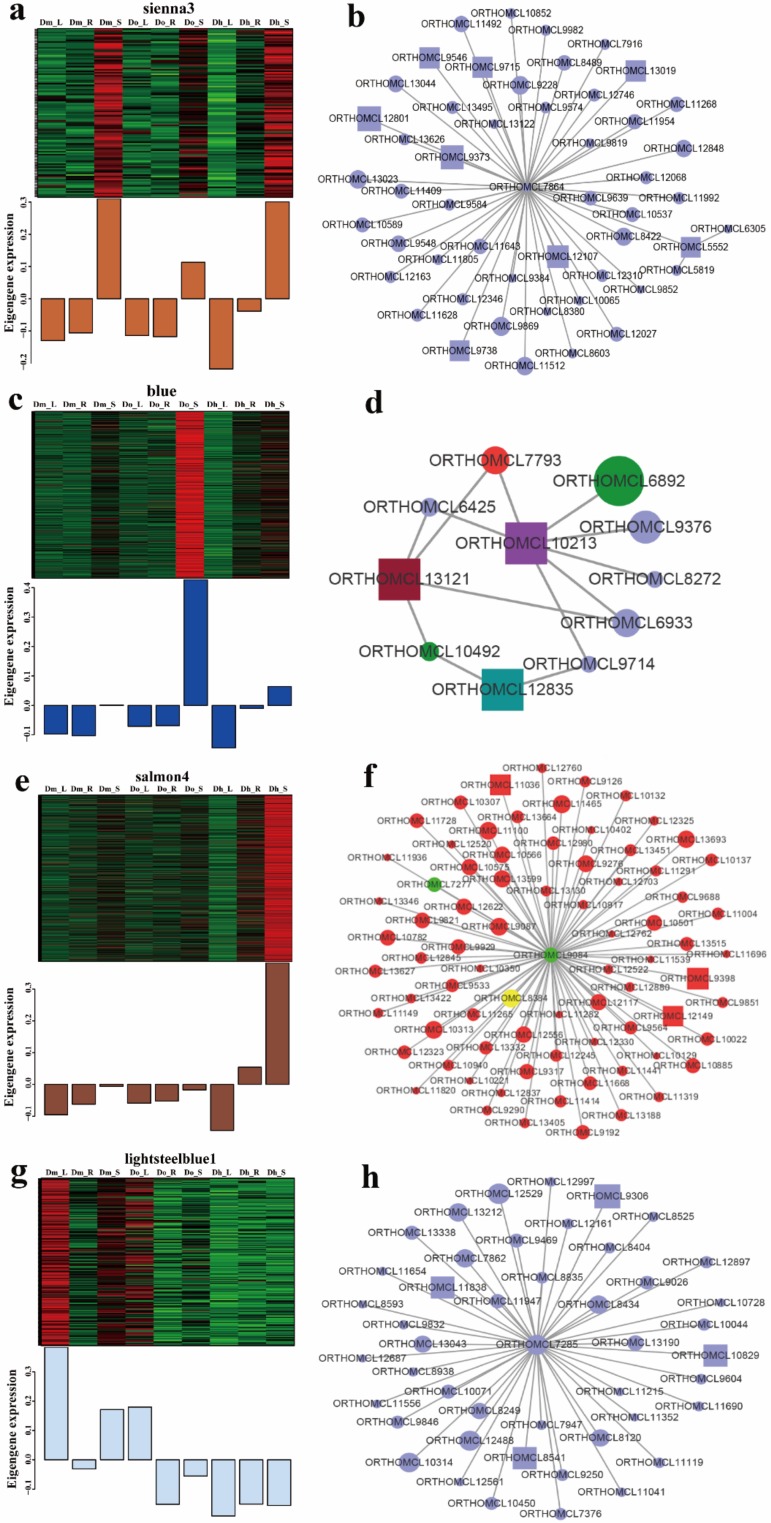
Expression profile and transcriptional regulatory network associated with the tissue-specific modules. (**a**), (**c**), (**e**), and (**g**) heatmaps showing genes in module that were significantly over-represented in Dm_L, Dm_R, Dm_S, Do_L, Do_R, Do_S, Dh_L, Dh_R, Dh_S and predicted transcriptional regulatory network associated with the gene sets showing expression patterns at Dm_L, Dm_R, Dm_S, Do_L, Do_R, Do_S, Dh_L, Dh_R, and Dh_S. Heatmaps show the expression profile of all the co-expressed genes (number given on the top) in the modules (labelled on top). Candidate hub genes are shown in rectangular shapes. Purple in figure (**b**, **d** and **h**) represents genes related to polysaccharides. Red in figure (**d** and **f**) represents genes related to secondary metabolites. The shapes, from big to small, indicate the weights from big to small. Green in figure d represents bHLH transcription factors. Green in figure (**f**) represents bZIP transcription factors and yellow represents MYB transcription factors.

We performed genes trend expression analysis of four modules of interest (Figure 12). Interestingly, by analyzing the trend expression of genes in sienna3 module, we found that they are specifically expressed only in the stem, which suggests that they play an important role in the stem. Through further analysis of this module, several hub genes were discovered, such as beta-glucosidase and granule-bound starch synthase (*GBSSI*). This indicates that the specific expression of these polysaccharide-related genes in stems may be related to the content of polysaccharides in stems significantly higher than in other tissues.

In the blue module, Do_S shows specific expression in this module, several hub genes were detected in each important module, with each gene interacting with many other genes. These genes include bHLH, beta-glucosidase, alpha-L-fucosidase, and other genes related to *Dendrobium* polysaccharide and secondary metabolism. In salmon4 module, Do_S shows specific expression in this module, hub genes include *CCR*4 (cinnamoyl -CoA reductase), bZIP, and MYB transcription factors were identified. In lightsteelblue1, a large number of genes were annotated to *ketohexokinase* (*KHK*). *KHK*, also known as fructokinase, which is a key enzyme in fructose metabolism. Fructose is a kind of plant polysaccharide, which suggests that this module is related to polysaccharide biosynthesis.

## 3. Discussion

Traditional biological research focuses on elucidating the effects of individual functional elements (such as DNA, mRNA, and protein) on life activities at the molecular level. Although those methods are of great significance for revealing the genetic mechanisms of specific traits, it can only partially explain the cause of a certain life activities. With the rapid development of sequencing technology, traditional biological research cannot fully and effectively explore the biological significance contained in massive data. As a research method of systems biology, the network is widely used in the exploration of life sciences with the help of data of genome, transcriptome, and metabolome. Compared with other regulatory networks, WGCNA can screen for genes related to specific traits and perform modular classification from large samples to obtain highly biologically significant co-expression modules, which has proven to be an efficient data mining method [35].

WGCNA has been widely used in plants in recent years. In order to obtain key expression modules and key hub genes related to drought resistance in *Brassica napus* L., WGCNA was used to analyze *Brassica napus* transcriptome data in multiple samples (48 transcriptome data), the well-watered and droughted networks contained 17 and 20 modules, respectively, suggesting that there are additional expression patterns in the droughted network because of rearrangement of the transcriptome in response to the drought treatment. [36]. In the study of *Fragaria* L. flowers [37], researchers generated different tissue- and stage-transcriptomic profiling of woodland strawberry (*Fragaria vesca*) flower development, they discovered a developing receptacle-specific module exhibiting similar molecular features to those of young floral meristems and hub genes of the strawberry homologs of a number of meristem regulators, including LOST MERISTEM and WUSCHEL in the developing receptacle network. [37]. Analysis of the pollen transcriptome of three male sterile lines using weighted gene co-expression network analysis revealed that two modules were significantly associated with male sterility and many hub genes that were differentially expressed in the sterile lines [38]. Farcuh et al. used WGCNA to investigate sugar metabolism during leaf and fruit development of two Japanese plum varieties, and identified 11 key sugar metabolism-related genes, the results showed that sugar metabolism was reprogrammed in a non-climacteric bud mutant of a climacteric plum fruit and showed an increase in sorbitol synthesis [39]. In *Ginkgo biloba*, a total of 12 gene modules were revealed to be involved in flavonoid metabolism structural genes and transcription factors by constructing co-expression networks, they reveal that some hub genes operate during the biosynthesis by identifying transcription factors (TFs) and structure genes and seven key hub genes were also identified by analyzing the correlation between gene expression level and flavonoids content [40]. Through these studies, it was found that many of the hub genes obtained by WGCNA analysis were indeed very important genes. In order to obtain the hub gene related to the synthesis and regulation of Dendrobium polysaccharides and alkaloids, we also performed WGCNA analysis.

Therefore, we constructed a *Dendrobium* gene co-expression network using a WGCNA approach and identified co-expression modules using transcriptome data from three kinds of *Dendrobium* species and three different tissues. Correlation analysis between co-expression modules and two traits (species and tissues) was carried out, and four highly significant active ingredients-related modules (*p*-value < 0.05) were identified. These modules consist of highly connected functional genes, and different modules appear to be involved in individual functions [41]. Meanwhile, KEGG pathway enrichment analysis of modules associated with polysaccharide and secondary metabolism indicated that these pathways in different *Dendrobium* species and different tissues are related to each other at the transcriptomic level. In sienna3 module, we found that several hub genes related to polysaccharide biosynthesis, such as *KHK* (*ketohexokinase*) which is a key enzyme in fructose metabolism. In the expression trend of this module, the stem has a clear advantage, indicating that the polysaccharide content is indeed concentrated on the *Dendrobium* stem, which is consistent with our previous determination of the polysaccharide content. Meanwhile, in blue module, we found that not only the hub genes related to polysaccharide, but also related to secondary metabolism. In the expression trend, we found that the stem of *Dendrobium officinale* to be the highest. This result is consistent with the trend in the determination of polysaccharides and alkaloids. The results also indicated that one component can be regulated by multiple modules, and one module can simultaneously be associated with multiple components.

Module hub genes are generally considered representative of a given module in a biological network. Previous studies reported that MYB-bHLH-WDR (MBW) ternary complexes comprise the essential regulatory machinery for catechin and anthocyanin biosynthesis [42]. In the present study, transcription factors MYB and bHLH were identified as hub genes in modules related to secondary metabolism. In addition, several genes involved in polysaccharide and secondary metabolism biosynthesis (*CCR4: cinnamoyl -CoA reductase* and *KHK: ketohexokinase*) were identified in modules. MYB transcription factors play an important role in the regulation of phenylpropane biosynthesis. Phenylpropane synthesis is upstream of the regulation of flavonoid biosynthesis, indicating that MYB is also an important transcription factor for the synthesis of flavonoids. In gentian, both GtMYBP3 and GtMYBP4 can activate the gene expression of flavonol synthesis, and then significantly increase the flavonol content in seedlings [43]. Ginkgo GbMYBF2 inhibits the expression of *CHS* (*chalcone synthase*), *F3H* (*flavanone 3-hydroxylase)*, *FLS* (*flavonol synthase*, Flavonol synthase and ANS genes) on the phenylpropane synthesis pathway, thereby reducing the content of flavonoids and anthocyanins [44]. *Salvia miltiorrhiza* SmMYB39 affects the synthesis of rosmarinic acid by regulating the expression of key enzyme genes of the phenylpropane metabolic pathway [45]. Current studies indicate that plant bHLH transcription factors are involved in regulating various signal transduction and anabolic pathways, such as light signal transduction, hormone synthesis, glandular and root hair development, and stress [46,47,48,49].

In this study, the WGCNA method was used for the first time in *Dendrobium*, and the modules related to specific tissues and genes related to specific traits were identified. Hub genes were further analyzed to find related genes and predict gene functions [30]. Combining the WGCNA method and RNA-Seq data can be used to better mine the genes and transcription factors related to traits. In strawberry, modules related to tissue specificity such as torus were found in strawberry, and 7 hub genes were identified in torus tissue [37]; in tomato, genes related to vitamin C biosynthesis were found [50]; co-expression modules related to acidity and genes related to anthocyanin synthesis were found in apple [33,51]. In addition, specific modules in other tissues such as roots, leaves, flowers, and fruits at other periods can be excavated to find relevant metabolic processes and important genes and potential transcription factors.

## 4. Materials and Methods

### 4.1. Plant Material

*Dendrobium* plants were cultivated artificially in a greenhouse located in Anhui Tongjisheng Biotechnology Company, Lu’an, China. Seed germination, the growth of protocorm-like bodies protocols and the condition of pots used for planting were described by our previous study [15]. Two-year-old *D. huoshanense*, *D. officinale,* and *D. moniliforme* were selected to provide three replicates of each sample. Stems, leaves, and roots were collected from three different kinds of *Dendrobium* plants for RNA extraction. Then, after drying, they were used to determine polysaccharide and total alkaloid contents in March, 2017. The same samples were used for transcriptome sequencing and polysaccharide and total alkaloid contents determination.

### 4.2. Polysaccharide and Total Alkaloid Contents Determination

The method for determining the polysaccharide content is consistent with the Chinese Pharmacopoeia (version 2010), and the details are described in our previous study [15]. The method of total alkaloids has been modified based on the Bush et al. method and has been described in detail in our previous studies [52,53]. A part of data of this part has been published [14,15].

### 4.3. Identification of Orthologous Genes

Datasets for three kinds of *Dendrobium* species were obtained from the NCBI Sequence Read Archive (SRA) (https://www.ncbi.nlm.nih.gov/sra) with accessing number SRP122499, SRP139000, and SRP150489. There was a total of 27 samples of *Dendrobium* that were taken from three different species (*Dendrobium huoshanense*, *Dendrobium officinale,* and *Dendrobium moniliforme*) and three different tissues (stems, leaves, and roots). The data for these samples were submitted by our own laboratory. The OrthoMCL pipeline [54] was used with standard settings to identify potential orthologous genes in *Dendrobium*, and subsequently aligned using the MUSCLE algorithm [55].

### 4.4. Differential Gene Expression Analysis

The gene expression levels of all samples were estimated by RSEM version 1.2.15 [56], and the bowtie2 parameter setting mismatch was 0. The R Bioconductor package edgeR [57] was used to identify differentially expressed genes (DEGs) in two samples. DEGs were used for KEGG and GO enrichment analyses, which were performed using the KOBAS version 2.0.12 with default settings and Goatools version 0.5.9 (https://github.com/tanghaibao/Goatools) [58,59].

### 4.5. WGCNA Analysis

#### 4.5.1. Construction of Weighted Gene Co-Expression Networks and Identification of Modules

We use thresholds for different expression data to calculate the number of genes. The WGCNA algorithm was applied to the evaluation of gene expression. The flashClust toolkit (R language) was used to perform cluster analysis on samples and set appropriate thresholds. After background correction and quantile normalization, the top 50% of genes (8056 genes) with the most variant in the analysis of variance were selected for WGCNA analysis. The WGCNA package in R was used to construct the co-expression network of the 8056 genes after verification [30].

The gradient independence method was used to test the scale independence and average connectivity of different power modules (the power value ranging from 1 to 20). According to the scale-independent conditions, the appropriate power value was 0.6. After the power value determined, the module was constructed by the WGCNA algorithm, and the genetic information corresponding to each module was extracted. For high reliability of the results, the minimum number of genes per module was set to 50.

#### 4.5.2. Interactions Analysis of Co-Expression Modules

The interaction relationship between different co-expression modules was calculated by WGCNA, and the topological overlap matrix (TOM) was constructed using the correlation expression values. The resulting topological overlap is a biologically meaningful measure of gene similarity based on the co-expression relationship between two genes. Using each TOM as input, the flashClust function was used for hierarchical cluster analysis. Then the dynamicTreeCut algorithm (minModuleSize = 30) was used to detect the module as a branch of the dendrogram [30]. Random colors were assigned to the modules, and the first principal component was used to calculate the module characteristic genes of each module. The module characteristic genes can be regarded as the representative of the gene expression pattern in this module, and the module combining highly relevant characteristic genes (merge-CutHeight = 0.07). By calculating the Pearson correlation between the module characteristic genes and the interest characteristics, and using the module characteristic genes to estimate the module-trait relationship, the samples are classified according to the corresponding traits, types, and tissues, and the module with a correlation coefficient ≥ | 0.75 | and *p*-value ≤ 0.01 was selected for further analysis. Through the heat map of the gene network topological overlap, the structure of co-expression module was visualized. Then through the hierarchical clustering tree diagram of the characteristic genes and the heat map of the corresponding characteristic gene network, the relationship between the modules was summarized.

#### 4.5.3. Functional Enrichment Analysis of the Key Module

We performed GO and KEGG enrichment analysis for key modules with most genes by using the Database for Annotation, Visualization and Integrated Discovery (DAVID, https://david.ncifcrf.gov/summary.jsp) [60] and KEGG Orthology Based Annotation System (KOBAS, https://bio.tools/kobas). Analysis results were extracted out under the condition of *p* < 0.05 after correction [61,62].

#### 4.5.4. Identification of Hub Genes

The hub gene is usually used as an abbreviation for a highly connected gene, which has a high degree of connectivity in co-expression modules. Module membership (MM) is defined as the correlation between the expression of each gene and its module eigengenes (MEs). Gene significance (GS) is defined as the correlation of each gene to the trait of interest. In the module of interest, the genes with the highest MM and GS were set as candidate genes for further analysis [63]. We chose the intramodular hub genes by external traits based GS > 0.2 and MM > 0.8 with a threshold of *p*-value < 0.05 [64]. In our study, based on the size of the module, we classified the top 10% of the most connected genes in the module as hub genes. We construct and visualize gene–gene interaction networks using Cytoscape tool [65].

## 5. Conclusions

Weighted gene co-expression network associated with traits was constructed, and 19 tissue and species-specific modules were obtained. The biological significance of the tissue and species-specific module was revealed, the regulatory genes related to the medicinal components of *Dendrobium* were identified, and a local biologically significant network was constructed. The results of this study can provide important experimental data and theoretical basis for the further analysis of the genetic mechanism of the medicinal ingredients of *Dendrobium*.

## Figures and Tables

**Table 1:** The number of genes in 19 constructed modules.

Module Colors	Node Number
Blue	562
Brown	892
Cyan	1132
darkgreen	1016
darkolivegreen	362
darkorange	665
floralwhite	184
greenyellow	440
lightcyan	180
lightgreen	178
lightpink4	40
lightsteelblue1	193
paleturquoise	470
plum2	57
royalblue	275
saddlebrown	179
salmon4	1015
sienna3	109
yellowgreen	107

## Supplementary Materials

Supplementary materials can be found at https://www.mdpi.com/1422-0067/21/3/861/s1.
