# Supplementary material for: Comparative Transcriptome Analysis of Different Dendrobium Species Reveals Active Ingredients-Related Genes and Pathways
Source: Int J Mol Sci. 2020 Jan 29;21(3):861. doi: 10.3390/ijms21030861 (PMC7037882; doi:10.3390/ijms21030861)
Supplement: Supplementary file 1 [file ijms-21-00861-s001.pdf]

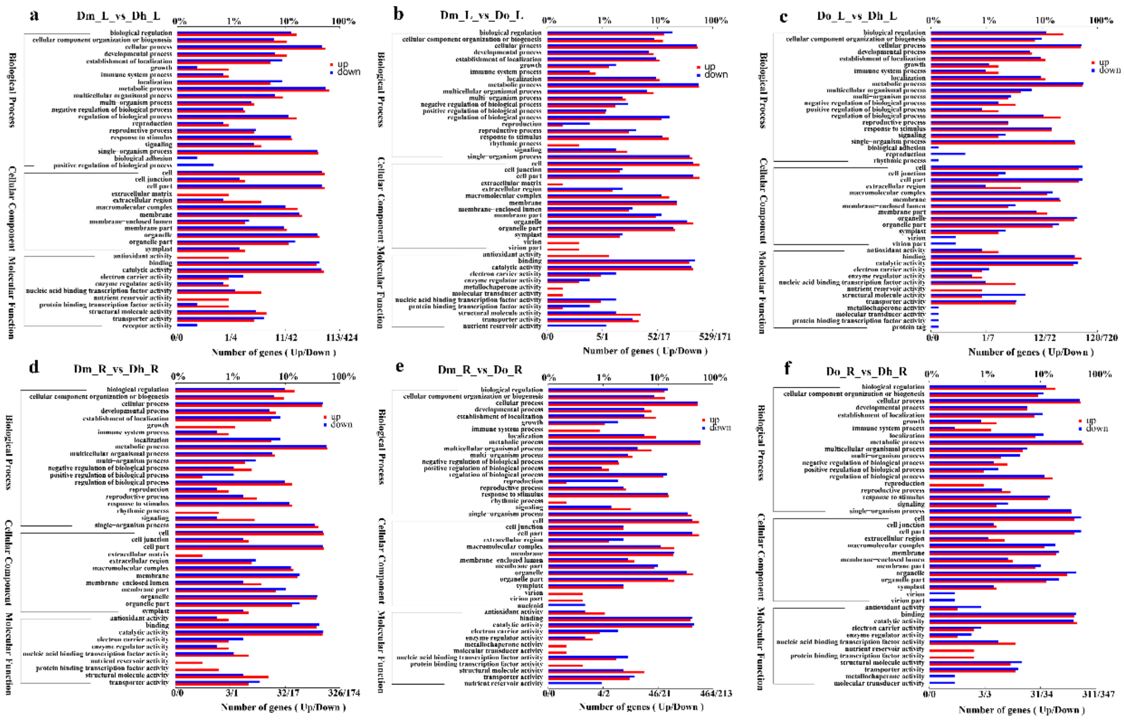

Supplementary figure 1 GO annotations of up and down regulated DEGs. The bottom x-axis indicates the number of DEGs annotated to a GO term, the upper x-axis indicates the proportion of DEGs annotated to a GO terms to the total number of all GO annotated DEGs; and the y-axis represents each detailed classification of GO. a. Dm_L vs Dh_L GO annotation; b. Dm_L vs. Do_L GO annotation; c. Do_L vs. Dh_L GO annotation; d. Dm_R vs Dh_R GO annotation; e. Dm_R vs. Do_R GO annotation; f. Do_R vs. Dh_R GO annotation.

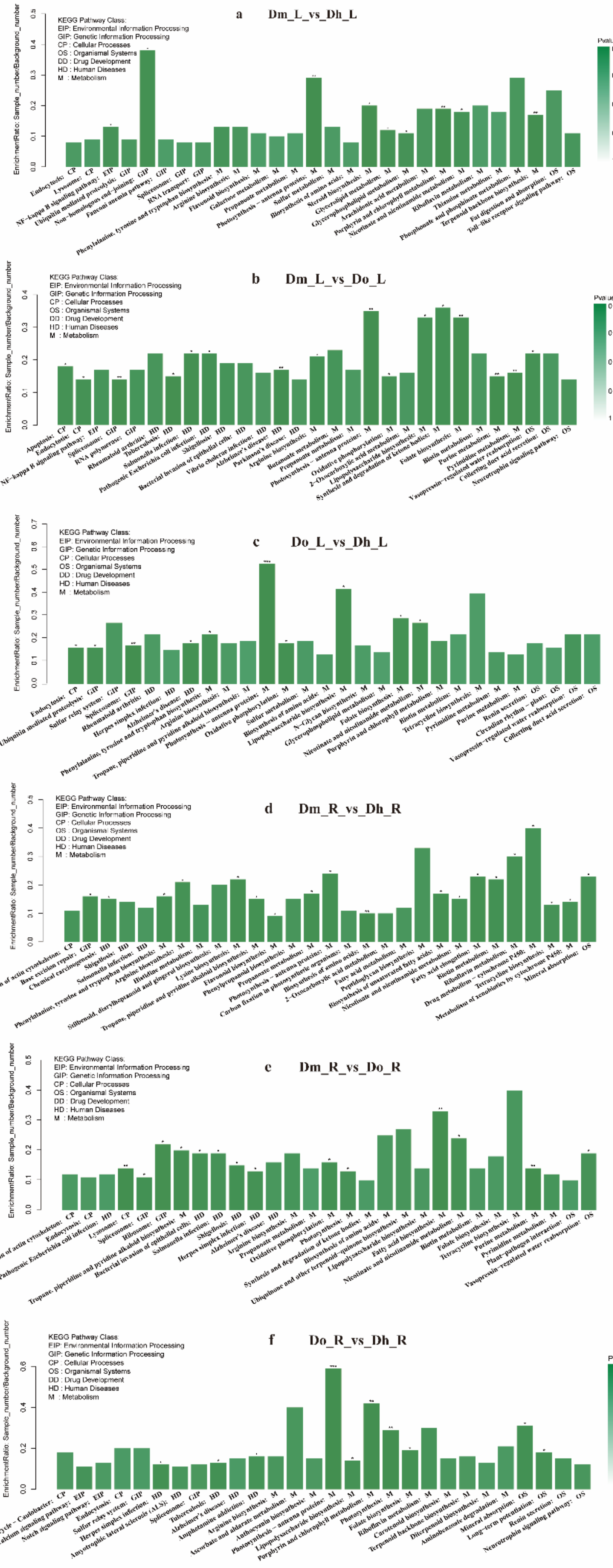
a Dm_L_vs_Dh_L
b Dm_L_vs_Do_L
c Do_L_vs_Dh_L
d Dm_R_vs_Dh_R
e Dm_R_vs_Do_R
f Do_R_vs_Dh_R
KEGG Pathway Class:
EIP: Environmental Information Processing
GIP: Genetic Information Processing
CP : Cellular Processes
OS : Organismal Systems
DD : Drug Development
HD : Human Diseases
M : Metabolism
EnrichmentRatio: Sample_number/Background_number
Pvalue

Supplementary Figure 2 Kyoto Encyclopedia of Genes and Genomes (KEGG) pathway enrichment of DEGs. The x-axis represents the pathway name, and the y-axis represents the enrichment ratio (sample number/background number). a. Dm_L vs. Dh_L; b. Dm_L vs. Do_L; c. Do_L vs. Dh_L; d. Dm_R vs. Dh_R; e. Dm_R vs. Do_R; f. Do_R vs. Dh_R. All pathways in the figure with asterisks indicate significant KEGG enrichment, with three asterisks indicating p-value < 0.001, two asterisks indicating p-value < 0.01, one asterisks indicating p-value < 0.05.
